# A comprehensive meta-review of systematic reviews and meta-analyses on nonpharmacological interventions for informal dementia caregivers

**DOI:** 10.1186/s12877-020-01547-2

**Published:** 2020-04-15

**Authors:** Sheung-Tak Cheng, Fan Zhang

**Affiliations:** 1 Department of Health and Physical Education, The Education University of Hong Kong, 10 Lo Ping Road, Tai Po, N.T Hong Kong; 2 Department of Clinical Psychology, Norwich Medical School, University of East Anglia, Norwich, UK

**Keywords:** Dementia caregivers, Intervention, Systematic review, Meta-analysis, Meta-review

## Abstract

**Background:**

Many reviews with conflicting findings on dementia caregiver interventions have been published. A meta-review was conducted to synthesize the findings of systematic reviews and meta-analyses.

**Methods:**

MEDLINE, PsycINFO, CINAHL and Cochrane Library were searched to identify reviews published during 2006–2018.

**Results:**

Sixty reviews covering > 500 intervention studies were selected and appraised with Assessment of Multiple Systematic Reviews (AMSTAR) II. The great majority of studies were of low quality according to AMSTAR II, but quality factors appeared unrelated to the conclusions obtained. Depression was most modifiable, with effects found across a spectrum of interventions (psychoeducation, counseling/psychotherapy, occupational therapy, mindfulness-based interventions, multicomponent interventions, etc.). Evidence of intervention effect was also found for quality of life (psychoeducation), mastery (psychoeducation, occupational therapy and multicomponent interventions) and communication skills (communication training). Null or weak results were found for anxiety, social support and burden. Support groups and respite were generally ineffective. There was no evidence that dyadic programs were better than caregiver-only programs, or that programs delivered individually or in groups would differ in their impacts. The evidence also does not support multicomponent interventions to have broader impacts than single-component programs. Methodological issues in the existing reviews (e.g., selective use of studies to serve different research purposes and inconsistent classification of interventions) were noted and taken into account when interpreting findings.

**Conclusions:**

This meta-review clarified variations in review methodology and identified a few potent groups of intervention (most notably psychoeducation, psychotherapy, occupational therapy, and multicomponent interventions), although no intervention type had broad effects on caregiver outcomes. We note that improvements are needed in the reporting of intervention studies and in making the classification of interventions more transparent and consistent. We further recommend fewer and larger-scale reviews and more attention to positive outcomes in order to better inform the field. Developing interventions with broader impacts and packaging them to meet caregivers’ changing needs in the course of dementia should be a priority for researchers and practitioners.

## Introduction

A report by the Alzheimer’s Disease International suggests that informal care accounts for approximately 40% of the annual care cost for dementia in high-income countries but 70–90% of the care cost in low- and middle-income countries [1]. In 2015, informal care provided at home to people with dementia amounted to 82 billion hours globally—equivalent to over 40 million full-time workers [2]. Such care is provided over many years given dementia’s chronic course, and the cumulative stress can have significant impacts on their physical and mental health (e.g., depression, anxiety, cardiovascular diseases, sleep disturbance) [3], thus interferring with their ability to sustain providing care. How to optimize support for informal caregivers has become a prominent issue for societies around the world. Against this context, it is imperative to know the types of intervention that are helpful to caregivers. This article provides a critical meta-review of this literature, focusing on systematic reviews and meta-analyses published in the last 13 years and on outcome variables including burden, depression, anxiety, quality of life (QoL), mastery, and social support.

A large number of studies have been conducted to evaluate interventions for dementia caregivers, and the literature has grown considerably in terms of quality and scope [4]. Notably, more recent studies have improved in scientific rigor (e.g., use of randomized controlled designs, monitoring treatment fidelity, power analysis, blinding participants and assessors, and assessing long-term outcomes) [4].

With the proliferation of the caregiver intervention studies, numerous reviews with varying quality have been published to synthesize the findings. In addition, 5 meta-reviews on intervention effectiveness have been published since 2015 [4–8], covering systematic reviews and meta-analyses published from 1988 to 2014, mostly after 2003. As an emerging tool, meta-review can be an important method to summarize a broad and heterogeneous literature [9]. As some researchers have argued, “meta-reviews, which pull together existing synthesis literature, can have tremendous influence on research, practice, and policy; indeed, if conducted appropriately, synthesis literature is considered the strongest level of evidence, with meta-reviews atop the evidence pyramid” [10].

The number of review articles covered by these meta-reviews ranged from 10 to 31, with a total number of 47 (after excluding duplicates and reviews without examining caregiver outcomes). Despite variations in research purpose and scope, a consistent message from the meta-reviews was that multicomponent interventions appeared to be most effective for reducing caregiver burden [4], maintaining caregiver health, and delaying institutionalization of the care-recipient (CR) [6]. It appears that the most effective component in multicomponent intervention is psychoeducation [6, 7], especially when combined with a therapeutic component [6]. Meanwhile, the effectiveness of intervention also varies across different caregiver outcomes [4, 7]; for example, Gitlin and Hodgson [4] noted that while multicomponent showed the best effect in reducing burden, relaxation training and psychoeducation worked better for lowering anxiety and depression respectively. Another conclusion found across meta-reviews was that interventions tailored to the needs of the individual caregiver tend to be effective [4, 5, 7]. However, the majority of existing interventions were relatively general, rather than targeting caregivers’ specific needs [8]. Moreover, four meta-reviews assessed the methodological quality of the review studies [5–8] and took this into account when interpreting findings.

A major issue in the existing meta-reviews, however, is the lack of consistency in the categorization of intervention programs. Gaugler and colleagues [11] have cogently pointed out how common it is for an intervention study to be classified differently across reviews, making it difficult to draw conclusions about which intervention is more effective. Therefore, instead of merely summarizing the reviews, it is necessary to look into the studies selected for different types of intervention.

In addition, none of the meta-reviews to date have provided a thorough coverage of the literature. Approximately half of the relevant literature was regularly left out by the previous meta-reviews for reasons not immediately apparent to this research team, even after considering their search strategies (details are available from the first author). Hence, together with the need to update the literature, a thorough meta-review of the literature is warranted. This meta-review covers the literature from 2006 to 2018 because a comprehensive meta-analysis of > 120 intervention studies was published in 2006 [12]. This meta-analysis was widely considered an authoritative summary of the literature up to 2005. Thus, a meta-review starting from 2006 would include this important meta-analysis as well as new reviews covering the period after 2005. By synthesizing the reviews on interventions for informal dementia caregivers, we aim to identify the most effective interventions for different caregiver outcomes, as well as identify the aspects that need to be improved in future research and practice.

This meta-review focuses on direct interventions for caregivers and so reviews concerning interventions for the CR, but without simultaneous caregiver involvement (i.e., not dyadic interventions), are not included even though they might cover caregiver outcomes as a result. Moreover, we will not include reviews focused on interventions for caregivers whose CRs were institutionalized as such reviews were too few to allow meaningful analysis.

## Methods

### Search strategy

Searches were conducted in three electronic databases: MEDLINE, PsycINFO and CINAHL with Full Text to identify relevant peer-reviewed systematic reviews or meta-analyses in English that were published between January, 2006 and December, 2018, including articles e-published ahead of print. Search terms were (dementia or Alzheimer* or “mild cognitive impairment” or MCI or “neurocognitive disorder*”) AND (carer* or caregiv* or “care partner*”) AND (review* or meta-analy*) AND (intervention* or trial* or treatment* or therap* or training or respite or “day care” or control or random*). In addition, the Cochrane Database of Systematic Reviews were searched. Whenever there was ambiguity in the title and abstract, the full text of the review article was read thoroughly to determine its suitability. The search was performed by the second author, and the results were double-checked by the first author. Any disagreement was resolved through discussions between the two authors. Articles meeting the inclusion/exclusion criteria were then selected for this meta-review.

### Inclusion and exclusion criteria

To be included, the article has to report systematic review and/or meta-analysis, with search strategy and inclusion/exclusion criteria clearly described. Also, intervention outcomes on informal caregivers must be included, although they do not have to be the exclusive focus of the review study. Informal caregivers include relatives, friends or neighbors providing community- or home-based care for individuals with dementia. Reviews not written in English or not published in peer-reviewed outlets were excluded. Reviews of pharmacological interventions, process evaluations of interventions, scoping reviews, and other topics (e.g., cost-effectiveness, service utilization, clinical translation) were excluded. Also excluded were direct interventions for the CR and the caregivers were not involved in delivering the interventions. We do not limit this meta-review to particular caregiver outcomes, and so reviews reporting a range of outcomes related to the caregivers’ functioning and well-being, including care competence, knowledge, self-efficacy, social support, relationship with the CR, stress, burden, depression, anxiety, quality of life, and resilience, were included for examination.

### Data extraction and synthesis

Data were extracted by the second author and double-checked by the first author. A form was designed to extract the following data: review author, year, country, search period, search database, studies reviewed, review method, sample characteristics, intervention approach, measured caregiver outcomes, and major findings. Grouping of interventions was done by both authors together through discussion until a consensus was reached.

### Quality assessment

The methodological quality of included reviews was assessed by the second author using the tool Assessment of Multiple Systematic Reviews (AMSTAR) II [13], which has a possible score of 0 to 18 summed from 16 items (see Table 1 footnote). 15% of the review studies were randomly selected for independent rating by the first author. The interrater reliability for the total number of items with flaws (i.e., zero scores) were good (*r* = 0.89, *k* = 13, *p* < 0.001, where *k* is number of reviews).

**Table 1:** Methodological quality ratings based on AMSTAR II (*k* = 60)

	AMSTAR II items^a^																	
	1	2^b^	3	4^c^	5	6	7^c^	8	9i^b^	9ii^b^	10	11i^b^	11ii^b^	12	13^b^	14	15^c^	16
Abrahams et al., 2018 [14]	1	0.5	0	0	1	1	0	0	1	9	0	0	9	0	0	1	0	1
Backhouse et al., 2017 [15]	1	0.5	0	0.5	1	1	0	0.5	0.5	9	0	0	9	1	1	1	1	1
Bernardo et al., 2018 [16]	0	0	0	0.5	0	0	0	0.5	0	0	0	9	9	9	0	0	9	0
Boots et al., 2014 [17]	0	0.5	0	0.5	1	1	0	1	1	0	0	9	9	9	1	0	0	1
Brodaty & Arasaratnam, 2012 [18]	0	0.5	1	0	0	1	0	0	1	1	0	1	1	1	0	0	1	1
Chien et al., 2011 [19]	1	0.5	0	0	1	1	0	0	1	0.5	0	0	0	0	0	1	1	1
Clarkson et al., 2018 [20]	1	1	1	0.5	1	1	0	0	1	0.5	0	9	9	9	0	0	9	1
Collins & Kishita, 2019 [21]	1	0.5	1	0.5	1	1	0	1	0	0	0	0	0	1	0	1	1	1
Cooper et al., 2007 [22]	0	0.5	0	0	0	1	0	0.5	0	0	0	9	9	9	0	0	0	0
*Corbett et al., 2012 [23]	0	0.5	0	0.5	1	0	1	1	1	9	0	1	9	0	0	0	9	1
Dam et al., 2016 [24]	1	0.5	0	0	1	1	0	1	0.5	0.5	0	9	9	9	0	0	9	1
Deeken et al., 2018 [25]	1	0.5	0	0	1	1	0	0.5	1	9	0	0	9	0	0	1	0	1
Egan et al., 2018 [26]	0	1	0	0	1	0	0	0.5	1	1	0	9	9	9	0	0	9	0
Eggenberger et al., 2013 [27]	1	0.5	1	0.5	1	1	0	0	0.5	0.5	0	9	9	9	0	0	9	1
Elvish et al., 2013 [28]	0	0	1	0	1	1	0	0.5	1	1	0	9	9	9	0	0	9	0
Gallagher-Thompson & Coon, 2007 [29]	0	0	0	0	1	0	0	1	9	9	0	9	9	0	0	0	9	0
Godwin et al., 2013 [30]	0	0.5	0	0	1	1	0	0	1	9	0	9	9	9	0	0	9	1
Greenwood et al., 2016 [31]	0	0.5	1	0.5	1	1	0	1	0	0.5	0	9	9	9	0	0	9	1
Hopkinson et al., 2019 [32]	1	0.5	1	0.5	1	1	0	1	1	1	0	0	0	1	1	1	1	1
Hurley et al., 2014 [33]	0	0.5	0	0.5	0	1	0	0.5	1	0.5	0	9	9	9	0	0	9	0
Jensen et al., 2014 [34]	1	0.5	0	0.5	1	1	0	0.5	1	9	1	0	9	1	1	9	1	1
Jütten et al., 2018 [35]	1	0	1	0	1	1	0	1	0	0	0	0	0	0	0	1	1	1
Kaddour et al., 2019 [36]	1	1	0	0.5	1	1	0	0.5	0	9	0	0	9	0	0	1	1	1
Kishita et al., 2018 [37]	1	0.5	0	0.5	1	1	1	0.5	0	9	0	0	9	0	0	1	0	1
Kor et al., 2018 [38]	1	0.5	1	0	0	1	0	0.5	1	1	0	0	0	0	1	1	0	1
Lamotte et al., 2017 [39]	1	0.5	0	0	0	0	0	1	0	0	0	9	9	9	0	0	9	0
Laver et al., 2017 [40]	1	0.5	0	0	1	1	0	0	1	9	0	0	9	0	0	1	0	1
Li et al., 2013 [41]	0	0.5	0	0.5	0	1	0	0.5	0.5	9	0	0	9	0	0	1	0	1
*Lins et al., 2014 [42]	1	0.5	0	0.5	1	1	1	1	1	0.5	1	1	1	0	1	1	0	1
*Liu et al., 2017 [43]	1	0.5	0	0	1	1	0	0.5	1	9	0	1	9	0	0	1	0	1
Llanque & Enriquez, 2012 [44]	0	0	0	0	0	0	0	0	0	0	0	9	9	9	0	0	9	1
Maayan et al., 2014 [45]	1	0.5	0	0.5	1	1	1	1	1	9	1	0	9	0	1	1	1	1
McKechnie et al., 2014 [46]	1	0.5	0	0	1	0	0	0.5	1	1	0	9	9	9	1	0	9	1
Morris et al., 2018 [47]	1	0.5	1	0.5	1	1	0	0.5	1	1	0	9	9	9	0	0	9	1
Nguyen et al., 2019 [48]	1	0.5	1	0	1	1	0	0.5	0.5	0.5	0	1	1	0	0	1	1	0
Olazarán et al., 2010 [49]	1	0.5	0	0.5	1	1	0	0	0	9	0	1	9	1	1	1	1	1
Orgeta et al., 2014 [50]	1	0.5	0	0	1	1	1	1	1	9	0	0	9	0	0	1	0	1
Parra-Vidales et al., 2017 [51]	1	0	0	0.5	1	0	0	0.5	0	0	0	9	9	9	0	0	9	0
*Petriwskyj et al., 2016 [52]	1	0.5	1	0.5	0	1	1	0.5	0.5	0.5	0	9	9	9	1	0	9	1
Piersol et al., 2017 [53]	0	0.5	1	0	1	1	0	0.5	1	1	0	9	9	9	0	0	9	0
Pinquart & Sörensen, 2006 [12]	0.5	0.5	1	0.5	1	1	0	1	0	0	1	1	1	0	0	1	0	1
Powell et al., 2008 [54]	0.5	0	0	0	1	1	0	0	0	0	0	9	9	9	0	0	9	0
Rausch et al., 2017 [55]	0	0.5	0	0.5	1	1	0	0.5	1	1	0	9	9	9	1	0	9	1
Schoenmakers et al., 2010 [56]	0.5	0.5	0	0.5	1	0	0	0.5	0.5	0.5	0	0	0	0	0	1	1	1
Scott et al., 2016 [57]	0	0.5	1	0.5	1	1	0	0.5	1	1	0	0	0	1	1	1	0	1
Selwood et al., 2007 [58]	0	0.5	0	0	0	1	0	0.5	0	0	0	9	9	9	0	0	9	1
Smith et al., 2014 [59]	0	0.5	0	1	0	0	0	1	0	0	0	9	9	9	0	0	9	1
Smits et al., 2007 [60]	0	0.5	0	0.5	1	1	0	0.5	1	1	0	9	9	9	0	0	9	0
Tang et al., 2016 [61]	0	0.5	0	0	1	0	0	1	0.5	0.5	0	9	9	9	0	0	9	1
*Thompson et al., 2007 [62]	0	0.5	0	0.5	1	1	0	0.5	1	9	0	1	9	1	1	0	0	1
Tretteteig et al., 2016 [63]	0	0.5	1	0	0	0	0	1	9	0	0	9	9	9	0	0	9	1
Tyack et al., 2017 [64]	0	0.5	0	0	0	1	0	0.5	0.5	0.5	0	9	9	9	0	0	9	1
Vandepitte et al., 2016 [65]	0	0.5	1	0.5	1	1	0	0.5	0.5	0.5	0	9	9	9	0	0	9	1
Vandepitte et al., 2016 [66]	1	0.5	1	0.5	1	1	0	0.5	1	1	0	9	9	9	1	0	9	1
*Vernooij-Dassen et al., 2011 [67]	0.5	1	0	1	1	1	1	1	0.5	9	0	1	9	0	1	1	1	1
Waller et al., 2017 [68]	0	0.5	1	0.5	1	1	0	0.5	1	1	0	9	9	9	1	0	9	1
Weinbrecht et al., 2016 [69]	0.5	0.5	0	0.5	1	1	0	0.5	1	9	0	0	9	0	0	1	0	1
Williams et al., 2019 [70]	1	0.5	1	0.5	0	0	0	0.5	1	9	0	0	9	0	0	1	1	1
*Wilson et al., 2017 [71]	1	0.5	0	0	1	1	0	0	0.5	0.5	0	9	9	9	1	0	9	1
Ying et al., 2018 [72]	1	0.5	0	0	1	1	0	0	1	1	0	9	9	9	0	0	9	1
Total^d^	35	53	20	33	46	47	7	47	43	28	4	9	4	8	17	24	14	48

0 = no; 0.5 = partial yes; 1 = yes; 9 = others, e.g., no meta-analysis. Articles with the publication year of 2019 were e-published within the search period

^a^For AMSTAR II items, 1 = covering PICO (participants, intervention, comparator group, outcome) in inclusion criteria; 2 = following review protocol; 3 = study design selection; 4 = literature search strategy; 5 = duplicated coding for study selection; 6 = duplicated coding for data extraction; 7 = justification of excluded papers; 8 = description of included studies; 9i = assessment of risk of bias (ROB) of RCT; 9ii = ROB of NRSI; 10 = reporting study funding source; 11i = using appropriate statistical combination method in RCT; 11ii = using appropriate statistical combination method in NRSI; 12 = ROB impact on meta-analysis; 13 = discussing of ROB impact; 14 = addressing heterogeneity; 15 = consideration of publication bias; 16 = reporting conflict of interest

^b^Critical domains; ^c^critical domains applicable for systematic reviews (without meta-analysis) only. The high- (weakness in ≤1 non-critical domain) or moderate- (weakness in ≥2 non-critical domains) quality reviews are marked by an asterisk

^d^The total number of “partial yes” and “yes” was summed

## Results

### Study characteristics

**Fig. 1:**
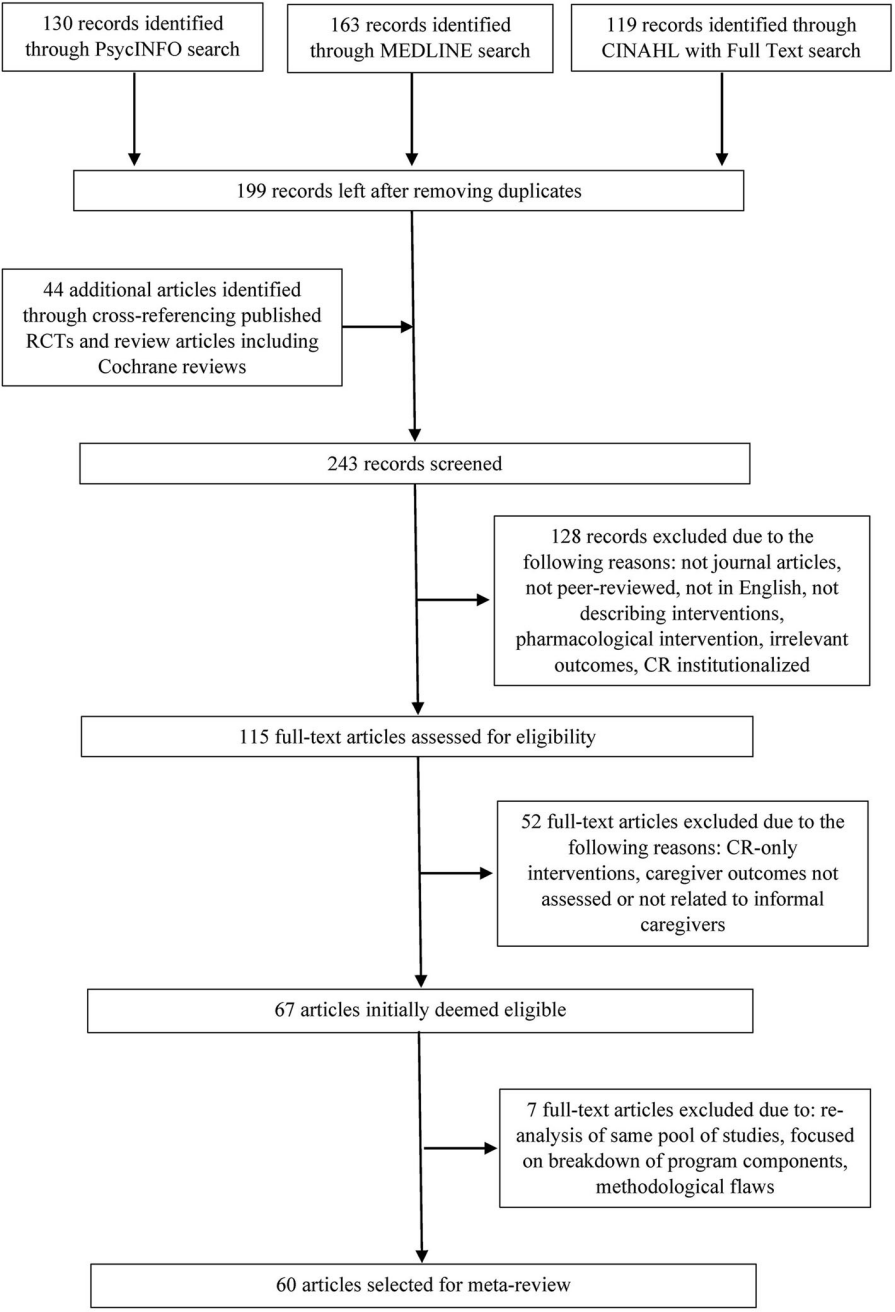
Flow diagram of the literature search

Two hundred forty-three titles and abstracts were screened and 115 papers were identified for full paper inspection, of which 67 were deemed eligible initially (see Fig. 1). Four reviews were further deleted because three were re-analysis of the same pool of studies by the same research teams, and one review was an attempt to break programs down to different components, without directly linking outcomes to the individual programs. In addition, we found three reviews with significant flaws (including removal of studies with nonsignificant effects from meta-analysis [73], misrepresenting *raw* mean differences as effect sizes [74], and misrepresenting effects with confidence intervals containing zeros as significant [75]), which were also excluded. Thus, 60 reviews were selected for this meta-review. Findings based on only one intervention study within each review do not appear in Table 2; thus this meta-review focuses on findings with a certain degree of replication, or lack of, at the time of publication of each of the reviews.

**Table 2:** Characteristics of review studies (*k* = 60)

Author (year)	No. of studies (search period)	Databases	Review method	Interventions	Major findings^a^
Abrahams et al., 2018 [14]	22 RCT (up to Sept, 2015)	CINAHL, MEDLINE, PubMed, PsycINFO, OTseeker, EMBASE, Cochrane Library	Systematic review, meta-analysis	Multicomponent interventions	Burden (− 0.23), depression (− 0.21), self-reported health (0.24), social support (0.26)
	Remarks: The authors claimed that only studies with CGs and CRs coresiding were included – a criterion clearly not met by the included studies. Moreover, not all interventions were clearly multicomponent.				
Backhouse et al., 2017 [15]	14 RCT (up to 2015)	MEDLINE, Cochrane Library, EMBASE, PsycINFO; HMIC, SPP, ProQuest, ICTRP	Systematic review, meta-analysis	Care coordination delivered by a single professional	Burden (− 0.54) ne: Mood, QoL, social support
Bernardo et al., 2018 [16]	8 RCT, NRSI and QS (2006–2015)	Web of Science, MEDLINE, CINAHL, PsycINFO and 4 other databases	Systematic review	Occupational therapeutic interventions with a focus on physical and social environmental modifications	↓ Burden, overload ↑ Sense of competence
	Remarks: 1 study focused on institutionalized CRs was excluded.				
Boots et al., 2014 [17]	12 RCT and NRSI (Jan, 1988-Jan, 2013)	PubMed, PsycINFO, CINAHL, Web of Science, Cochrane Library	Systematic review	Internet-based interventions	↓ Burden, depression ↑ Sense of competence, self-efficacy
Brodaty & Arasaratnam, 2012 [18]	23 RCT and NRSI (no period specified) on interventions with CR BPSD as outcome	MEDLINE, EMBASE, PubMed, PsycINFO, Scopus	Meta-analysis	Community-based non-pharmacological interventions for CR delivered with involvement of CG	Overall beneficial effect on a range of outcomes (e.g., burden, behavioral bother, self-efficacy, and confidence in managing BPSD) (0.15)
Chien et al., 2011 [19]	30 RCT and NRSI (1998–2009)	MEDLINE, Cochrane Library, PubMed	Systematic review, meta-analysis	Support groups and psychoeducation^b^	Burden (−0.23), depression (− 0.40), mental health (0.44) Social outcomes (inc. social support, QoL and relationship with CR) (0.40)
	Remarks: The theme of the review was support group interventions but > 80% of the interventions were psychoeducation groups.				
Clarkson et al., 2018 [20]	68 RCT, NRSI and QS (up to Apr, 2014)	PubMed, Cochrane Library, PsycINFO, CINAHL and 2 other databases	Systematic review	Home support programs (inc. education, advice, behavioral management, psychological support, environmental modification, and care coordination)	↓ Burden ↑ Well-being
	Remarks: 2 studies solely focused on CRs were excluded.				
Collins & Kishita, 2018 [21]	12 RCT and NRSI (up to Dec, 2016)	PsycARTICLES, PsyINFO, MEDLINE Complete, Scopus, Web of Science, ProQuest	Meta-analysis	Mindfulness- and acceptance-based interventions	Depression (post-intervention − 0.98, follow-up − 0.71), burden (post-intervention − 0.66, follow-up − 0.53)
Cooper et al., 2007 [22]	24 RCT and NRSI (up to June, 2005) with CG anxiety as outcome	Allied and Complementary Medicine, British Nursing Index, CINAHL, EMBASE, MEDLINE, PsycINFO	Systematic review	Group CBT	↓ Anxiety
				Behavioral management techniques	ne: Anxiety
				Exercise interventions	ne: Anxiety
				Multicomponent interventions	ne: Anxiety
				Respite	ne: Anxiety
				Relaxation and yoga	↓ Anxiety
	Remarks: Psychoeducational interventions with CBT components were also included in the group CBT category. One of the two studies on relaxation and yoga [76] was in fact a psychoeducational program with 17% of the time allocated to progressive muscle relaxation and autogenic training.				
Corbett et al., 2012 [23]	13 RCT (2001–2008)	Cochrane Library, EMBASE, MEDLINE, PsycINFO	Systematic review, meta-analysis	Provision of information and advice	ne: Burden/stress QoL (0.36)
Dam et al., 2016 [24]	29 RCT, NRSI and QS from 39 articles (Jan, 1988-May, 2015	PubMed, PsycINFO, CINAHL, Web of Science, Cochrane Library	Systematic review	Support groups, befriending/peer support, mobilizing informal support networks, remote support interventions^B^	↑ Social support, social satisfaction ne: Burden, depression, self-esteem, QoL
	Remarks: Family counseling programs were also included. Support interventions often omitted social support outcomes. Evidence on improved social support was derived mainly from qualitative studies. Based on quantitative studies, support groups had no effect, whereas remote support and network mobilization (inc. family counseling) interventions yielded mixed results.				
Deeken et al., 2018 [25]	33 RCT (up to Aug, 2018)	PubMed, PsycINFO, Cochrane Library	Meta-analysis	Multimodal technology-based interventions	Burden (− 0.20), depression (− 0.31) Inconclusive: Behavioral bother
				Telephone intervention	ne: Burden Depression (−0.24)
				Computer/web-based interventions	ne: Burden, depression
				All interventions combined	Burden (−0.13), depression (− 0.20)
Egan et al., 2018 [26]	8 RCT (up to Sept, 2016)	PubMed, Cochrane Library, CINAHL, Web of Science, PsycINFO, EMBASE	Systematic review	Internet-based interventions	↓ Depression, anxiety, stress ne: Behavioral bother, knowledge, coping, QoL Inconclusive: Self-efficacy
Eggenberger et al., 2013 [27]	4 RCT and NRSI (up to Jan, 2010)	MEDLINE, AMED, EMBASE, PsycINFO, CINAHL, Cochrane Library, Gerolit, Web of Science	Systematic review	Communication skills interventions	↓ Communication problems ↑ Skills and knowledge about communication
	Remarks: 8 studies focused on institutionalized CRs were excluded.				
Elvish et al., 2013 [28]	20 RCT, NRSI and QS (2005–2011) on psychological interventions	MEDLINE, PsycINFO, ERIC, PubMed	Systematic review	Psychoeducation with-skill building	↓ Depression ↑ Well-being, QoL
				Multicomponent interventions	↓ Depression ↑ Social support, well-being
				Technology-based	↓ Burden, depression ↑ Social support, well-being
Gallagher-Thompson & Coon, 2007 [29]	19 RCT and NRSI (1980–2005) on psychological interventions	MEDLINE, PsycINFO, ERIC, PubMed	Systematic review	Psychoeducation with skill building	Distress (−0.81)
				CBT-based psychotherapy and counseling	Distress (−1.20)
				Multicomponent interventions	Depression (−0.33)
Godwin et al., 2013 [30]	5 RCT from 8 articles (1990-May, 2012)	Medline, PsycINFO, EBSCO	Systematic review	Technology-driven interventions	↓ Depression, anxiety ne: Social support
	Remarks: 3 studies based on the same RCT were merged, and 1 meta-analytic study was removed.				
Greenwood et al., 2016 [31]	4 RCT and NRSI (up to 2014)	MEDLINE, EMBASE, Cochrane Library, PsycINFO, CINAHL Plus and 2 other databases	Systematic review	Multicomponent interventions delivered by healthcare professionals	↓ Burden, depression
	Remarks: All interventions were delivered by healthcare providers in general practice.				
Hopkinson et al., 2019 [32]	25 RCT and NRSI (up to Jan, 2017)	MEDLINE, EMBASE, CINAHL, PsycINFO, Cochrane Library	Systematic review, meta-analysis	CBT	Depression (post-intervention − 0.34; follow-up − 0.99), stress (post-intervention − 0.36); effects driven by group programs only ne: Anxiety, QoL
	Remarks: Psychoeducation and multicomponent interventions with CBT components were included, as was a study testing psychodynamic group psychotherapy.				
Hurley et al., 2014 [33]	8 RCT and NRSI (2004–2012)	PsycINFO, MEDLINE, Scopus, EMBASE	Systematic review	Meditation-based interventions	↓ Burden, depression
Jensen et al., 2015 [34]	7 RCT (Feb, 2010-Feb, 2013)	MEDLINE, EMBASE, PsycINFO, CINAHL, AgeLine, CENTRAL, ERIC	Systematic review, meta-analysis	Educational interventions	Burden (− 0.52), depression (− 0.37) Inconclusive: QoL
Jütten et al., 2018 [35]	60 RCT and NRSI (Jan, 2002-Jan, 2017)	MEDLINE, PsycINFO, PsycARTICLES, Psychology and Behavioral Science Collections, Cochrane Library, EMBASE	Meta-analysis	Nonpharmacological interventions in general but excluding dyadic interventions, respite, case management and nursing interventions^b^	Burden (− 0.20), stress (− 0.18), depression (− 0.19), QoL (0.36), sense of competence (0.31) ne: Anxiety
Kaddour et al., 2019 [36]	14 RCT from 12 articles (up to July, 2017)	PsycINFO, MEDLINE, CINAHL, Scopus, Open Grey, ProQuest and 3 other databases	Meta-analysis	Low-intensity CBT-based interventions	Burden (− 0.53), distress (− 0.33), depression (− 0.27), anxiety (− 0.35)
	Remarks: Psychoeducation and multicomponent interventions with CBT components were also included. One intervention [77] branded as psychoeducation in Kishita et al. [37] was classified as CBT-based intervention in this review.				
Kishita et al., 2018 [37]	30 RCT (2006–2016)	MEDLINE, PsycINFO, Scopus, Cochrane Library	Systematic review, meta-analysis	Psychoeducation with-skill building	Burden (− 0.18) ne: Depression, QoL
				CBT-based psychotherapeutic interventions	ne: Burden Depression (− 0.15), anxiety (− 0.38)
	Remarks: Psychoeducation and multicomponent interventions with CBT components were also included.				
Kor et al., 2018 [38]	5 RCT (1990–2016)	MEDLINE, CINAHL, Cochrane Library, PsycINFO, EMBASE, Web of Science	Systematic review, meta-analysis	Mindfulness-based interventions	Depression (− 0.62), stress (− 0.57)
Lamotte et al., 2017 [39]	3 RCT and NRSI (1990–2016)	PubMed, Cochrane Library, EMBASE, Google Scholar	Systematic review	Dyadic exercise interventions	↓ Burden (no effect in one RCT)
	Remarks: 1 study without CG outcome was excluded.				
Laver et al., 2017 [40]	38 RCT (2008-Oct, 2015)	MEDLINE, EMBASE, PsycINFO	Systematic review, meta-analysis	CG-only interventions	ne: Burden, depression, QoL
				Dyadic interventions	ne: Burden, QoL Depression (− 0.33)
				All interventions combined	Behavioral bother (−0.26), QoL (0.24)
	Remarks: 2 studies focused on institutionalized CRs were excluded. No significant differences were found between dyadic and CG-only interventions. Moreover, all interventions, inc. psychoeducation, support groups, family counseling, etc. were regarded as multicomponent by the authors. We dropped the term “multicomponent” to avoid confusion.				
Li et al., 2013 [41]	8 RCT (up to July, 2011) on whether coping mediated intervention effects	EMBASE, MEDLINE, PsycINFO, Web of Science, Cochrane Library, CINAHL, AMED	Systematic review, meta-analysis	Group coping skills intervention	Depression (−0.91), dysfunctional coping (− 0.39)
				Group coping skills intervention with behavioral activation	Depression (−0.30), positive coping (0.28), dysfunctional coping (− 0.26)
				Remotely delivered intervention	↓ Depression (no pooled effect size) ne: Coping Inconclusive: Anxiety
Lins et al., 2014 [42]	11 RCT, NRSI and QS (May, 2011-Feb, 2013)	ALOIS, Cochrane Library	Systematic review, meta-analysis	Telephone counseling	Depression (−0.32) ne: Burden, self-efficacy, social support
				Telephone counseling with video sessions and a workbook	Inconclusive: self-efficacy
Liu et al., 2017 [43]	7 RCT (up to Apr, 2017)	Cochrane Library, MEDLINE, PsycINFO, EMBASE, CINAHL	Systematic review, meta-analysis	Mindfulness-based interventions	Depression (−0.58), stress (− 0.33), QoL (0.38) ne: Burden, anxiety
Llanque & Enriquz, 2012 [44]	9 RCT and NRSI (2000–2011) on interventions for Hispanic CGs	Google Scholar, Social Gerontology, Health Source: Nursing/ Academic Edition, MEDLINE, PsycARTICLES, CINAHL, PubMed	Systematic review	Psychoeducation (with psychotherapeutic components), family therapy, telecommunication interventions, telephone support groups, multicomponent interventions, etc.^b^	↓ Depression
	Remarks: 1 study without CG outcome was excluded.				
Maayan et al., 2014 [45]	4 RCT (1989–2009)	ALOIS, Cochrane Library	Systematic review, meta-analysis	Respite service providing home support	ne: Depression
	Remarks: It is noteworthy that respite was found to have no effect on any outcome but other outcomes are not listed here because they were assessed only once.				
McKechnie et al., 2014 [46]	14 RCT and NRSI (Jan, 2000-Sept, 2012)	MEDLINE, PsycINFO, CINAHL Plus	Systematic review	Computer-mediated interventions	↓ Burden, depression, anxiety ne: Self-reported health, social support Inconclusive: Mental health, self-efficacy
	Remarks: Reported results based on a subset of high- and medium-quality studies only.				
Morris et al., 2018 [47]	18 RCT, NRSI and QS (from 2010)	CINAHL Plus, MEDLINE, PsycINFO	Systematic review	Communication skills training	↓ Burden, depression, anxiety ↑ Knowledge, communication skills
Nguyen et al., 2019 [48]	11 RCT and NRSI (period not specified)	MEDLINE, EMBASE, CINAHL, ProQuest, PsycINFO	Systematic review, meta-analysis	Communication skills training	↑ Communication ↓ Psycho-physiological states, inc. stress, burden, distress, depression and anxiety (pooled effect sizes not provided separately for informal CGs)
	Remarks: 6 studies focused on institutionalized CRs and/or formal CGs were excluded.				
Olazarán et al., 2010 [49]	91 RCT (up to Sept, 2008)	MEDLINE, PsycINFO, CINAHL, EMBASE, Lilacs, Cochrane Library	Systematic review, meta-analysis	Psychoeducation (individual sessions)	ne: Mood
				Psychoeducation (group sessions)	ne: Mood, well-being
				Support via electronic devices	ne: Mood
				Multicomponent interventions	ne: Mood, well-being, QoL
	Remarks: 108 studies of CR-focused interventions were excluded.				
Orgeta et al., 2014 [50]	4 RCT (1997–2001)	MEDLINE, EMBASE, PubMed, PsycINFO, Scopus, Cochrane Library	Systematic review	Interventions for CG physical activity	Burden (−0.22) ne: Depression, anxiety, stress
Parra-Vidales et al., 2017 [51]	7 NRSI and QS (2010–2015)	PubMed, PsycINFO, Scopus, SciELO, Psicodoc	Systematic review	Online psychoeducation	↓ Depression, stress ↑ Self-efficacy/competence, knowledge ne: QoL
Petriwskyj et al., 2016 [52]	3 RCT, NRSI and QS (1990–2015)	CINAHL, PsycINFO, Web of Science, PubMed, EMBASE and 6 other databases	Systematic review	Interventions promoting resilience	ne: resilience
Piersol et al., 2017 [53]	36 RCT and NRSI (Jan, 2006-Apr, 2014)	MEDLINE, PsycINFO, CINAHL, OTseeker, Cochrane Library	Systematic review	Case management	↓ Burden ↑ Well-being
				Group interventions	↓ Burden, distress ↑ Well-being, self-efficacy, mental health
				CBT-based interventions	↓ Depression, anxiety, stress
				Psychoeducation	↓ Burden, depression ↑ QoL, well-being, self-efficacy
				Other single-component interventions (e.g., mindfulness-based, communication skills training, exercises programs)	↓ Burden/stress, depression, guilt ↑ Communication skills, well-being, mental health, hope, self-efficacy, relationship with CR
	Remarks: 7 papers reporting systematic reviews or meta-analyses were excluded. The category “psychoeducation” was originally labeled idiosyncratically as “multicomponent psychoeducational intervention” but the word “multicomponent” is dropped here to avoid misunderstanding. Group interventions included support groups, family meetings, etc.				
Pinquart & Sörensen, 2006 [12]	123 RCT and NRSI (1982–2005)	PsycINFO, MEDLINE, Ageline, PsyINDEX	Meta-analysis	Psychoeducation with CG active participation	Burden (−0.20), depression (− 0.36), SWB (0.21), ability/knowledge (0.55)
				Psychoeducation-information only	Knowledge/ability (0.28) ne: Burden, depression, SWB
				CBT	Burden (−0.36), depression (− 0.70) ne: SWB, knowledge/ability
				Counseling/case management	Burden (−0.50) ne: Depression, knowledge/ability, SWB
				Support interventions	ne on outcomes (except one study with an effect on well-being)
				Respite	Burden (−0.26), depression (− 0.12), SWB (0.27) ne: Knowledge/ability
				Multicomponent interventions	ne on any outcome (except for institutionalization of CR)
				Miscellaneous (e.g., life review)	ne on any outcome
	Remarks: 4 studies solely focused on CRs were excluded.				
Powell et al., 2008 [54]	15 RCT and NRSI (up to Aug, 2007)	MEDLINE, EMBASE, CINAHL, PsycINFO, AMED	Systematic review	Technology-based interventions	↓ Burden, depression
Rausch et al., 2017 [55]	5 RCT and NRSI (up to Apr, 2016)	PubMed, PsycINFO, CINAHL	Systematic review	Dyadic interventions inc. group reminiscence, art viewing, art making; training individualized activities	↑ Mood, confidence (QS only)
	Remarks: 2 studies without CG outcomes were excluded.				
Schoenmakers et al., 2010 [56]	26 RCT and NRSI (1980–2007)	MEDLINE, EMBASE, Cochrane Library and 2 other databases	Systematic review, meta-analysis	Home care programs (inc. psychosocial interventions, respite, telephone support, and case management)	ne: Burden, depression (except for respite which was found to increase burden)
Scott et al., 2016 [57]	4 RCT and NRSI (from 1995)	PsycINFO, Cochrane Library, Scopus, MEDLINE	Systematic review, meta-analysis	Technology-based CBT	Depression (−0.21)
	Remarks: Psychoeducation and multicomponent interventions with CBT components were also included.				
Selwood et al., 2007 [58]	62 RCT and NRSI (up to July, 2003) on psychological interventions	Cochrane Library	Systematic review	Educational interventions	ne: Burden/stress, psychological health
				Group training in coping skills	↓ Burden, depression
				Individual training in coping skills	↓ Distress, depression
				Group behavior management techniques (BMT)	ne: Burden, depression, or distress
				Individual BMT (< 6 sessions)	ne Burden, depression, or distress
				Individual BMT (≥6 sessions)	ne: Burden ↓ Depression (both post-intervention and follow-up)
Smith et al., 2014 [59]	4 RCT, NRSI, and QS (up to Jan, 2013)	MEDLINE, EMBASE, PsycINFO, Social Policy and Practice, CINAHL Plus and 2 other databases	Systematic review	Peer support and befriending delivered by volunteers on an one-on-one basis	ne: Mental health, loneliness
Smits et al., 2007 [60]	25 RCT and NRSI (1992–2005)	MEDLINE, PsycINFO, EBM Reviews-Cochrane Library	Systematic review	Dyadic interventions	Inconclusive: Burden, depression, mental health, competence
Tang et al., 2016 [61]	14 RCT, NRSI and mixed-method (1995–2014)	CINAHL EBM Reviews, EMBASE, MEDLINE, PsycINFO and 3 other databases	Systematic review	Psychosocial interventions with self-efficacy as outcome	↓ Burden ↑ Self-efficacy
Thompson et al., 2007 [62]	44 RCT and NRSI (Nov. 2003-Oct, 2005) on information and support interventions^b^	Cochrane Library, MEDLINE, EMBASE, PsycINFO, CINAHL, SIGLE, ISTP, INSIDE and 17 other databases	Systematic review, meta-analysis	Technology-based support interventions	ne: Depression
				Support groups	ne: Burden
				Group psychoeducation	ne: Burden Depression (−0.71)
				Individual psychoeducation	ne: Depression, self-efficacy
Tretteteig et al., 2016 [63]	19 NRSI, QS or mixed-method (up to 2013)	PubMed, Norwegian Electronic Health Library, AMED, EMBASE, MEDLINE, PsycINFO	Systematic review	Day care services	↓ Burden, overload, depression ↑ Perceived support Inconclusive: Well-being
Tyack & Camic, 2017 [64]	7 RCT, NRSI, QS and mixed-method (period not specified)	PsycINFO, ASSIA, MEDLINE, CINAHL, Cochrane Library	Systematic review	Touchscreen (interactive computer-based) interventions	Inconclusive: Burden, well-being, relationship with CR
	Remarks: 9 studies without informal CGs were excluded.				
Vandepitte et al., 2016 [65]	17 RCT and NRSI (up to Jan, 2015)	PubMed, Web of Science	Systematic review	Respite	Inconclusive: Burden ne: Stress
Vandepitte et al., 2016 [66]	53 RCT and NRSI (up to Mar, 2015)	Web of Science, PubMed	Systematic review	Psychoeducation	↓ Burden, depression ↑ Self-efficacy
				Respite	Inconclusive: Burden
				Occupational therapy interventions	↑ Self-efficacy
				CBT	↓ Depression, dysfunctional thoughts
	Remarks: Psychoeducation and multicomponent interventions with CBT components were also included in the CBT category.				
Vernooij-Dassen et al., 2011 [67]	11 RCT (up to Apr, 2009)	Cochrane Library, MEDLINE, EMBASE, PsycINFO, CINAHL and 2 other databases	Systematic review, meta-analysis	Cognitive reframing	Depression (−0.24), anxiety (− 0.21), stress/distress (− 0.24) ne: Burden, behavioral bother, coping/self-efficacy
	Remarks: CBT and psychoeducation were both included and analyzed together.				
Waller et al., 2017 [68]	34 RCT and NRSI (Jan, 1990-Dec, 2016)	MEDLINE, EMBASE, CINAHL, Cochrane Library	Systematic review	Computer (inc. tablet, website, e-mail or mobile app) interventions	↓ Burden/stress, depression ↑ Mental health, knowledge, positive aspects of caregiving
				Telephone (inc. text messaging, telehealth, videophone) interventions	Inconclusive: Burden, depression, mental health, managing BPSD ne: Social support, self-efficacy, health, self-care
				Multimodal (computer plus telephone) interventions	Inconclusive: Burden, depression, self-efficacy
Weinbrecht et al., 2016 [69]	33 RCT (2005–2014)	MEDLINE, EMBASE, CENTRAL, PsycINFO, PsyINDEX	Meta-analysis	A range of intervention types (e.g., education, counseling, support group, communication skills, environmental modification, care coordination)^B^	Depression (post-intervention −0.13, follow-up − 0.29)
Williams et al., 2019 [70]	34 RCT (1999–2018) with burden as outcome	MEDLINE, PsycINFO, CINAHL	Systematic review, meta-analysis	Multicomponent interventions	Inconclusive: Burden
				Education/skills-based interventions	Inconclusive: Burden
				Support and counselling	Inconclusive: Burden
				Physical activity	ne: Burden
Wilson et al., 2017 [71]	3 RCT and NRSI (from 1995-Sept, 2016)	MEDLINE, CINAHL Plus, EBSCO, PubMed, EMBASE, PsycINFO and 4 other databases	Systematic review, meta-analysis	Interventions targeting CG grief	↓ Grief (2 out of 3 studies)
Ying et al., 2018 [72]	15 RCT (up to Jan, 2017)	PubMed, EMBASE, Cochrane Library, Web of Science, EBSCO, PsycINFO	Systematic review	Multicomponent interventions	↑ Competence, knowledge, coping Inconclusive: Burden
	Remarks: Some CG-only interventions showed larger effects than dyadic programs.				

For the number of intervention studies included in each review, we eliminated those that did not report informal CG outcomes and made a remark beneath each study. Pooled effect sizes were not available for some meta-analytic studies as they were not reported by the study authors for various reasons. *BPSD* behavioral and psychological symptoms of dementia, *CBT* cognitive-behavioral therapy, *CG* caregiver, *CR* care-recipient; *NRSI* non-randomized study of intervention, *QoL* quality of life, *QS* qualitative study, *RCT* randomized controlled trial, *SWB* subjective well-being; ↑ = enhanced; ↓ = reduced

^a^Numeral in parenthesis refers to standardized or weighted mean difference when a significant effect was found; *ne* = no effect observed through either quantitative or qualitative synthesis. In terms of meta-analytic results, only pooled effects with *p* < 0.05 are reported as significant in the table, disregarding the interpretation provided in the original review article

^b^Effects were discussed or analyzed without differentiating types of intervention, or without categorizing interventions

The effectiveness of psychoeducation was addressed in 14 reviews [12, 23, 28, 29, 34, 37, 41, 49, 51, 53, 58, 62, 66, 70], counseling and psychotherapy – 10 reviews [12, 22, 29, 32, 36, 37, 53, 57, 58, 66], occupational therapy (OT) interventions – 2 reviews [16, 66], mindfulness-based interventions (e.g., mantra repetition, meditation, yoga, mindfulness-based stress reduction) – 5 reviews [21, 22, 33, 38, 43], support interventions (e.g., support groups, mobilizing informal networks) – 5 reviews [12, 24, 49, 59, 62], communication skills training – 3 reviews [27, 47, 48], respite – 6 reviews [12, 22, 45, 63, 65, 66], home care/support – 2 reviews [20, 56], care coordination and case management – 2 reviews [15, 53], physical activity interventions – 2 reviews [50, 70], and multicomponent interventions – 9 reviews [12, 14, 22, 28, 29, 31, 49, 70, 72]. Moreover, 4 articles provided reviews of dyadic interventions (involving caregiver and CR together) [39, 40, 55, 60] while 15 covered technology-based (e.g, telephone, internet) interventions [17, 22, 25, 26, 28, 30, 41, 42, 46, 51, 54, 57, 62, 64, 68]. In addition, 12 “miscellaneous” reviews covered themes that cannot be classified into any of the above categories (e.g., counseling merged with case management, and interventions targeting resilience or grief) [12, 18, 19, 22, 40, 44, 52, 53, 61, 67, 70, 71]. Two provided general overviews [35, 69]. Note that the above lists are not mutually exclusive as reviews often cover multiple categories of intervention or a specific class of interventions that, by nature, cross categorization boundaries (e.g., online support groups), and hence the total number does not add up to 60.

The categories of support interventions and counseling/psychotherapy deserve further consideration. The term “support intervention” has been used quite liberally in the literature. While a mutual support group of caregivers is the prototype of this kind of intervention, the term is sometimes used by researchers to refer to any intervention that has the potential of facilitating social support, including different group-based interventions and family counseling. An extreme case would be Chien et al.’s review of 30 “support groups” [19] which included 25 *psychoeducational* groups but only 5 support groups. While these interventions may enhance social support because of the contact provided, it was not the interventions’ primary aim. Moreover, other group approaches, such as group psychotherapy and group meditation, may be subsumed under this umbrella concept, with the distinct features of the different interventions masked. Thus, we classified Chien et al. under the miscellaneous category instead. We accept another review [24] with this issue being a minor one (affected studies constituting ≤15% of the total number of included studies).

Three reviews focused on cognitive-behavioral therapy (CBT) or CBT-based (psychotherapeutic) interventions [32, 36, 37] were all published or e-published in the same year with *few overlapping studies*. What all these reviews shared in common, however, were the fact that a substantial proportion of the interventions included were psychoeducational in nature (i.e., with structured content and delivery using a heavily educational, rather than psychotherapeutic, approach) and included CBT techniques in one or more of their modules. These interventions were recently recognized as *psychoeducation with psychotherapeutic components* [78, 79], a subtype of psychoeducation distinguished from others that do not borrow from psychotherapeutic approaches. Other reviews would have classified these interventions under the category of psychoeducation or psychotherapy. In this meta-review, we grouped these reviews together with others focused on counseling/psychotherapy on the basis that the psychoeducational interventions also shared a CBT theoretical framework, but readers should bear this issue in mind when interpreting the results. On the contrary, about half of the studies in a review of “cognitive reframing” interventions [67] were based on the stress-and-coping framework [80, 81], without clear connection to psychotherapeutic principles. This review was grouped under the miscellaneous category.

Note, however, that similar concerns about classification, or the consistency of classifications from one study to another, were not specific to reviews of support or counseling/psychotherapeutic interventions. Where such concerns exist, they are noted beneath the description of each study in Table 2. This information will be used to qualify conclusions drawn from the reviews.

Among the 60 included reviews, 32 (53%) were systematic reviews, whereas 28 (47%) were meta-analyses (mostly in conjunction with a systematic review). Twenty (33%) reviews included only randomized controlled trials (RCTs), whereas 38 (63%) included both randomized and non-randomized (mainly quasi-experimental) studies. Two (3%) reviews included only non-randomized studies, along with mixed-method and/or qualitative studies.

The number of studies covered by the reviews ranged from 3 to 123 (mean = 21.82, SD = 22.39, median = 14). Specifically, 20% (*k* = 12, where *k* is number of reviews) of the reviews were based on ≤5 studies, 15% (*k* = 9) on 6–10 studies, 22% (*k* = 13) on 11–15 studies. 8% (*k* = 5) on 16–20 studies, 15% (*k* = 9) on 21–30 studies, and 20% (*k* = 12) on > 30 studies. Thus, nearly two-thirds of the reviews based their conclusions on 20 or fewer studies, and over one-third relied on 10 or fewer studies.

A total of 555 nonoverlapping intervention studies were covered by all the reviews. It is noteworthy that among these 500+ studies, as many as 52% were included in only *one* of the reviews, despite their topical overlap. For those cited more than once, however, inconsistent classifications across reviews were not unusual.

About two-thirds of the reviews (*k* = 41, 65%) were conducted by authors based in Europe, especially UK (*k* = 23, 37%). Additionally, 6 reviews (10%) were based in US, 9 (14%) in Australia, 6 (10%) in East Asia, and 1 (2%) in Brazil. Caregivers were usually above 50, female, and being the adult child or spouse of the CR.

**Fig. 2:**
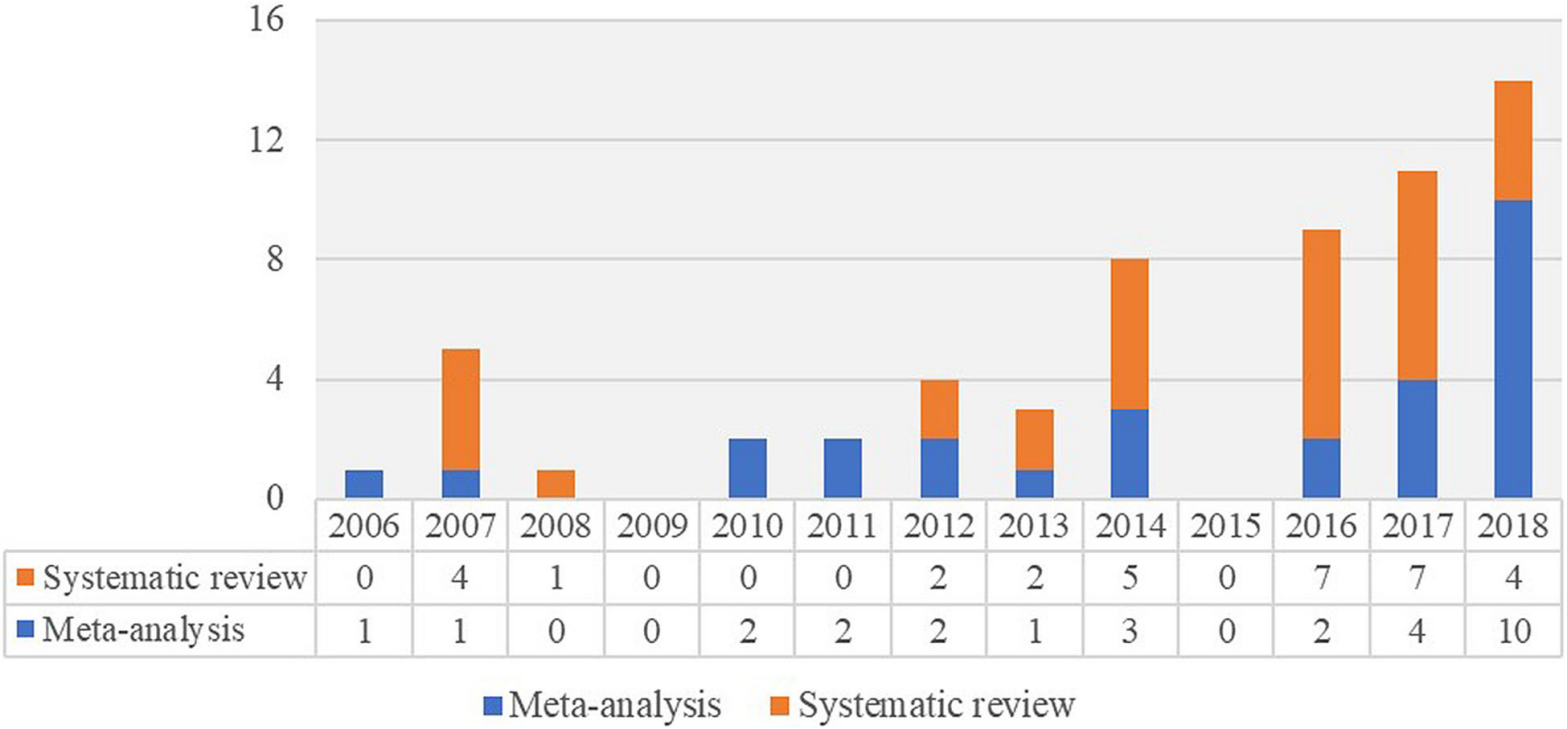
Number of reviews on dementia caregiver interventions (excluding reviews focused on interventions to train care-recipients directly or for caregivers with care-recipients residing in institutions) published between 2006 and 2018

Finally, there have been an increasing number of reviews published since 2014. In 2018, there was a sharp increase in the proportion of reviews that conducted meta-analyses (Fig. 2). It is not clear whether this is a continuing trend. The increase in the number of reviews provides a large “database” for this meta-review.

### Methodological quality

The average AMSTAR II score was 8.6 (range = 1–15). All but 7 (12%) [23, 42, 43, 52, 62, 67, 71] were rated as low quality (Table 1). The majority of moderate-quality reviews (none rated as high) were meta-analyses in the categories of psychoeducational and psychotherapeutic interventions. Across the reviews, the lowest score was found in “funding source report;” only 4 reviews provided the funders of individual studies. “Exclusion justification” was also neglected in most of the reviews, as only 7 provided a list to justify the reasons for excluding each study. In addition, among 28 reviews including meta-analyses, 32% explcitly stated pooling study results with appropriate weighting techniques, and 50% investigated publication bias and its impact on findings. Items receiving generally high scores (i.e., > 75% of the studies meeting the expectation) were “the presence of a priori plan for conducting review,” “having duplicate coders for study selection,” “having duplicate coders for data extraction,” and “providing details of included studies.”

### Intervention effects

Some reviews lumped a variety of outcomes together in their analyses [18, 19, 48]. Some outcome variables, most notably mental health, mood and distress were indicated by a diverse set of measures. For example, across reviews, “distress” encompassed depression, anxiety, anger and even burden. “Mental health” was indexed by a variety of symptom measures including depression and anxiety, but sometimes vitality, role functioning, social engagement, and even coping as well. The results pretaining to these outcomes are difficult to interpret because of the heterogeneous measures subsumed under each construct. Other outcomes including loneliness, grief and guilt were covered too irregularly to draw meaningful conclusions. In the following, we focus on the more commonly reported outcomes, including burden, depression, anxiety, QoL, mastery, and social support. Assessment of positive outcomes such as mastery provides a different lens to look at the ways by which interventions have made a difference in caregivers’ lives.

As aforementioned, there were 14 reviews that grouped interventions in peculiar ways or simply did not differentiate between different kinds of intervention. In the interest of space, these reviews will not be given detailed attention. Furthermore, results pertaining to technology-based interventions are difficult to interpret because the results could vary due to the medium of delivery (e.g., computer, internet, with or without interaction) or the program content. Besides, whether certain types of intervention (e.g., psychoeducation, CBT, support group) are especially suitable for technology-based delivery have not been given attention in the literature. While we will cover these reviews, we will not discuss in detail the variation in findings.

Finally, before proceeding, we need to highlight two important issues. A meta-review depends on the information provided by the existing reviews and their quality. However, reviews on the same topic often relied on an overlapped pool of studies and so the information contributed by these studies was repeated multiple times. The problem is further compounded by the same research teams (or research teams with shared members) conducting multiple reviews on the same or similar topics *at the same time*. To illustrate this issue, note that Vandepitte and colleagues [65, 66] published two reviews in 2016, both analyzing respite interventions using an overlapped set of studies (Table 2).

Relatedly, the two reviews mentioned earlier on CBT-based interventions, by Kishita and colleagues [36, 37], were also published or e-published in the same year (i.e., 2018). Although the two reviews had different research questions and inclusion/exclusion criteria, it is still not clear to the present authors why some studies appeared in one review, but not the other, and why the latter publication [36] did not mention the earlier one [37]. Furthermore, one intervention [77] classified as psychoeducation in one of these reviews [37] was branded as CBT-based intervention [36] in the other. Although we make use of these publications to illustrate the issue, it is by no means specific to these reviews. As a further example, multicomponent interventions were often classified into a variety of categories, depending on the review focus (e.g., a multicomponent interventions with components of telephone counseling and support group may be classified as technology-based intervention, counseling, or support group, as long as the category suits the review’s theme). Thus, some intervention studies, by virtue of being repeated across reviews, would contribute more heavily to the outcome of this meta-review, while others, because of inconsistent classifications across reviews, would contribute information to more than one intervention category. Readers should bear these issues in mind when reading the results.

#### Burden

Among the 43 reviews that analyzed burden (including measures of overload, stress and behavioral bother), over half (*k* = 28) provided support for intervention effects. Burden was found to be reduced by psychoeducation [12, 34, 37, 51, 53, 58, 66], counseling/psychotherapy [12, 32, 36, 53] OT interventions, [16], mindfulness-based interventions [21, 33, 38], communication training [47], respite/day care [12, 63], home support [20], care coordination/case management [15, 53], physical activity interventions [50], multicomponent interventions [14, 31], dyadic interventions [39], technology-based interventions [17, 28, 46, 51, 54], and miscellaneous or intervention programs in general [12, 19, 35, 53, 61].

At the same time, 20 articles provided negative or mixed reviews. These included reviews on psychoeducation [12, 23, 58, 62, 70], CBT-based psychotherapeutic interventions [37], mindfulness [43], support interventions [12, 24, 62], respite [65, 66], home care [56], physical activity interventions [70], multicomponent interventions [12, 70, 72], dyadic interventions [40, 60], technology-based interventions [25, 26, 42, 64, 68], and miscellaneous (e.g., physical activity, dyadic) interventions [40, 67, 70]. Note that the same article might have provided both positive and negative reviews, depending on the intervention category concerned.

Several observations need to be made. First, there was a tendency for qualitative reviews to conclude beneficial intervention effects, whereas meta-analyses yielded mixed results. Second, although one 2006 review found respite to relieve burden [12], no reviews since then have provided an updated support for these programs. Third, it is noteworthy that in one large-scale review [12], only those psychoeducational programs with active caregiver participation (e.g., role plays, skills rehearsal, home exercises) were found to reduce burden, but programs that involved primarily passive reception of information were not. Similarly, another extensive review [58] observed that merely educational interventions had no effect on burden, but psychoeducation with coping skills training in groups did.

On the whole, a variety of interventions have been found to reduce burden, but the evidence is quite mixed. Counseling/psychotherapy appeared to receive more consistently positive reviews, but the common inclusion of psychoeducational programs (with psychotherapeutic components) in these reviews complicates the interpretation. Support groups did not receive any positive reviews, whereas respite and technology-based interventions tended to yield more inconsistent reviews than other interventions. In meta-analyses that found an effect, the pooled effect size was typically small (~ 0.20), including a sizable meta-analysis of psychosocial interventions in general [35].

#### Depression

Forty-one reviews examined depression as an outcome. Of these, 35 found support for the interventions, including psychoeducation [12, 28, 34, 41, 51, 53, 58, 62, 66], counseling/psychotherapy [12, 32, 36, 37, 53, 58, 66], mindfulness [21, 33, 38, 43], communication training [47], respite/day care [12, 63], multicomponent interventions [14, 28, 29, 31], dyadic interventions [40], technology-based interventions [17, 25, 26, 28, 30, 41, 42, 46, 51, 54, 57], and miscellaneous/general interventions [19, 35, 44, 67, 69]. Only 11 articles provided negative/mixed reviews, covering psychoeducation [12, 37, 62], counseling/psychotherapy [58], support interventions [12, 24, 62], respite [45], home care [56], physical activity interventions [50], multicomponent interventions [12], dyadic interventions [60], technology-based interventions [62, 68], and miscellaneous (e.g., dyadic) interventions [12, 40].

Thus, there was widespread support from the existing reviews for the efficacy of interventions in reducing caregiver depression, with psychoeducation, counseling/psychotherapy, mindfulness, and multicomponent interventions receiving the strongest support. Again, no favorable reviews were found for support interventions. Given the mixture of findings and the relatively small number of intervention studies and reviews in other categories, it is premature to draw conclusions about the other types of intervention. Again, effect sizes, when significant, tended to be small, hovering around − 0.30.

#### Anxiety

In contrast to burden and depression, anxiety was covered in only 13 reviews (22%), probably due to the fact that few intervention studies have assessed this outcome. Of these, 7 found beneficial effects of interventions, including counseling/psychotherapy [22, 36, 37, 53], communication training [47], technology-based interventions [26, 30], and miscellaneous interventions (e.g., mixing progressive muscle relaxation with other techniques) [22]. By contrast, 7 articles, covering CBT, behavior therapy, mindfulness-based, physical activity, multicomponent, technology-based, and general interventions, found no effect on anxiety [22, 32, 35, 41, 43, 46, 50]. This included Jütten et al.’s meta-analysis [35], which found null effect for psychosocial interventions in general. It is noteworthy that three meta-analyses of CBT-based interventions led to contrasting conclusions, with Kishita and colleagues [36, 37] finding significant intervention effects in the region of − 0.35, whereas Hopkinson et al. [32] did not, most probably because the two groups of researchers had only *two studies* in overlap out of a total of 17 studies covered. Considering also that 3 of the 7 positive reviews were about technology-based or miscellaneous (e.g., exercise) interventions, the evidence does not speak to favorable intervention effect on caregiver anxiety.

#### Quality of life

QoL (including well-being) was covered by 17 reviews. A positive effect on QoL was found by 8 reviews on psychoeducation [12, 23, 28, 35, 53], mindfulness [43], respite [12], home support [20], case management [53], multicomponent interventions [31], and miscellaneous interventions [53]. Meanwhile, null or inconclusive results were reported by 10 reviews on psychoeducation [12, 34, 37], CBT [12, 32], support interventions [24], day care [63], multicomponent interventions [12], technology-based interventions [26, 51, 64], and miscellaneous (e.g., dyadic) interventions [40]. To summarize, there appears to be some support for psychoeducation on enhancing caregiver QoL, but the effects of the other interventions are too early to tell.

#### Mastery

By mastery, we refer to several interrelated constructs including ability, sense of competence and self-efficacy, which altogether were covered by 13 reviews. Eight reviews concluded that certain interventions could enhance mastery, including psychoeducation [51, 53, 66], OT interventions [16, 66], multicomponent interventions [72], technology-based interventions [17, 51], and miscellaneous/general interventions [35, 53, 61]. Five other reviews, however, found no effect of psychoeducation [62], technology-based interventions [26, 42, 46], or miscellaneous interventions [67]. Only 3 meta-analyses have been attempted on this outcome [35, 42, 62], with only one finding a significant effect size of 0.31 (sense of competence) for psychosocial interventions in general [35]. Weighing the balance, there appears to be emerging evidence supporting the efficacy of psychoeducation and OT interventions, and possibly multicomponent interventions, in enhancing caregiver mastery. Nevertheless, it is not clear whether any effect of psychoeducation is specific to the group format, as a meta-analysis [62] found no effect on self-efficacy for individual psychoeducation (unfortunately, a separate analysis for group psychoeducation was not provided).

#### Social support

Social support outcomes were covered in 10 reviews, of which 5 were positive and 5 were negative. Surprisingly, only one review of support interventions examined this outcome and found beneficial intervention effect [24]. The other positive reviews concerned day care [63], multicomponent interventions [14, 28], and technology-based interventions [28]. On the contrary, one review concerned with care coordination [15] and four reviews concerned with technology-based interventions [30, 42, 46, 68] did not find intervention effect on social support. A review focused on various types of support intervention [24] was particularly revealing, which found positive intervention effect on social support mainly in qualitative studies, whereas results from quantitative studies (especially those on support groups) were negative or inconsistent. On the whole, there is no clear evidence suggesting that interventions make a difference in caregivers’ social support, but note also that multicomponent interventions did not receive any negative review, yet.

## Discussion

This meta-review provides a comprehensive summary and assessment of the review literature on dementia caregiver interventions since 2006. Different from earlier meta-reviews, which simply summarized the findings of different reviews, we also examined reviews for potential methodological flaws and identified classification-nomenclature issues. As well, by virtue of including a large pool of reviews that together covered > 500 intervention studies, we were able to provide a more sensitive assessment of the evidence concerning different kinds of intervention. We focused on conclusions drawn from multiple studies to strengthen the evidence being amassed.

We found consistent support across reviews for various types of nonpharmacological interventions to reduce caregiver depression, and emerging evidence for enhancing mastery and QoL. However, there was little evidence for intervention effects on anxiety and social support, and mixed or weak evidence for burden. It may be that whereas depression is more modifiable, burden, anxiety and social support (for which external conditions may play a heavier role) are less so.

In terms of intervention types, we found evidence for psychoeducation (reducing depression and enhancing mastery and QoL), counseling/psychotherapy (reducing depression), mindfulness-based interventions (reducing depression), OT interventions (enhancing mastery), and multicomponent interventions (reducing depression and possibly enhancing mastery). Little beneficial effects have been found for respite, especially in randomized studies [45]; in fact, using respite sometimes may even arouse caregiver guilt [82].

Scholars have pointed out that the effects of interventions may be domain-specific [4, 12]. Thus, mindfulness-based interventions, an emerging approach to help caregivers, have been found to reduce depression. OT interventions, which often involve home visits to teach skills tailored to the home environment and the specific needs of the CR/caregiver, are prone to have a direct impact on mastery. Likewise, three reviews focused on communication skills training [27, 47, 48], though not covered specifically in results, showed overall enhancement in communication knowledge and skills, and decrease in communication problems between caregivers and CRs (Table 2).

It was not surprising to find counseling/psychotherapy to be effective for depression. However, as a number of reviews on CBT included psychoeducational programs with modules on CBT skills, results cannot be interpreted unambiguously [83]. It should be noted that these psychoeducation programs are quite different from CBT in a number of ways. As a hybrid approach, CBT content is usually delivered in a standardized manner via an educational approach to a small group of caregivers together, with a de-emphasis on the therapeutic relationship between the trainer and the caregiver. In fact, various types of lay people or paraprofessionals, rather than professional therapists, are usually recruited as trainers, although these trainers may work under the supervision of a professional. Moreover, these psychoeducational programs are best characterized as providing CBT principles and techniques to facilitate self-help by caregivers, rather than directly applying CBT to modify caregivers’ thoughts and behaviors [78]. Therefore, merging these psychoeducation programs with CBT obscures important differences between them. In fact, it is not sure whether the beneficial effects noted for psychoeducation in general were due in large part to these programs with psychotherapeutic components [83]. Supporting this speculation were results showing that only those psychoeducation programs with caregivers actively involved in the practice and application of skills [12], epitomized by programs with psychotherapeutic components, were effective for caregiver burden, depression and well-being. To arrive at more refined conclusions, future reviews should endeavor to distinguish between these two types of program.

It is generally believed that, by virtue of their more comprehensive nature, multicomponent interventions are more helpful to caregivers than single-component programs [4]. This meta-review only partially supports this contention; multicomponent interventions were found to have beneficial effects on depression and possibly mastery, but there was little support for their effects on burden, anxiety and QoL. Any conclusion about their effect on social support is probably premature despite two positive reviews. These results cast doubt as to whether multicomponent programs are a good way to address the multiple needs of caregivers. Nevertheless, it is important to note that multicomponent interventions are a heterogeneous set of programs, and their effectiveness depends on the exact components included and whether the components match the needs of the caregivers. The present pool of reviews did not examine further whether certain types of multicomponent programs were more effective than others.

A topic of concern is whether group or individual setting plays a role in intervention effectiveness. Although the two delivery formats were not directly compared against each other in intervention studies, two reviews on psychological interventions attempted to addressed this question. Selwood et al. [58] noted that behavioral management techniques, when coached in groups, were not effective, but the same techniques, when coached individually for six sessions or more, reduced depression. However, Hopkinson et al.’s (2019) meta-analysis found that CBT’s effects on stress and depression existed in group programs only. Perhaps it is easier to tailor individual programs to the specific needs of the caregiver, whereas group programs may offer other benefits (e.g., a sense of universality, mutual role models) unavailable to individual programs. Note, however, that group programs can also be delivered in such a way that the needs of each caregiver are addressed more personally. How to further enhance the effectiveness of group programs is an important question to pursue in future research as they can serve more caregivers and are more economical than individual programs.

Another topic of concern is whether delivering assistance to both caregivers and CRs would be more beneficial than involving the caregiver alone. Laver et al. [40] found no significant difference between the two approaches in terms of reducing caregivers’ reaction to CR’s behavioral problems.

The foregoing discussion suggests the importance of matching interventions to caregivers’ needs [78], and to select outcomes sensitively vis-à-vis what the intervention is intended to achieve. Recruiting the wrong participants (e.g., non-depressed caregivers for a CBT program) or measuring inappropriate outcomes may result in failures in detecting intervention effects. For example, Dam et al. [24] noted that many support interventions did not assess social support as an outcome. Likewise, whether an intervention is found to enhance competence or self-efficacy may depend on the domain assessed. However, both of these issues were rarely given attention in the existing reviews. Hence, it is not possible to speculate further to what extent these issues have affected the findings.

### Review quality

A few observations are made about assessing the methodological quality of reviews. First, we note that some of the AMSTAR II criteria might not have been required by journals at the time of publication of the reviews (e.g., explaining why only English articles were included, reporting funding source for each included study) and hence the score is biased toward the low end. Four critical domains, namely, justifying study exclusion, using appropriate statistical methods (i.e., weighting technique and heterogeneity adjustment), considering ROB impact, and investigating publication bias, got generally low ratings. Among them, the first domain may be overly challenging, especially for larger reviews, as it requires stating the exclusion reasons for each excluded study. Clarkson et al. [5] also remarked that certain items on AMSTAR might have overemphasized reporting style at the expense of methodological quality rating. As for the other three critical domains with generally low scores, we did not find the conclusions to systematically vary between reviews rated 0 or 1 on these domains.

Second, AMSTAR II rating tends to favor meta-analysis and Cochrane review. In our results, 6 out of 7 reviews with moderate quality were meta-analyses, and 3 were Cochrane reviews. At the same time, there was a tendency for AMSTAR II to favor reviews of a smaller scope because some of the requirements (e.g., providing reasons for exclusion, study by study) would be a lot more challenging for bigger reviews. As a matter of fact, the 7 moderate-quality reviews screened an average of 79 full-text articles and included an average of 12 studies (2 reviews included only 3 studies). Thus, the moderate-quality reviews were mostly based on small samples of studies (note that the number of studies for any particular outcome would most likely be just a subset of the total number covered in each review), whereas many sizable reviews covering a substantial proportion of the literature were rated low in quality. It is difficult to strike a balance between representativeness of the literature and methodological quality in this case.

Third, beyond AMSTAR, we examined the impact of including non-randomized or qualitative studies in the reviews, as these designs tend to yield more positive findings [12, 24]. Because the effects of randomized and non-randomized/qualitative studies were almost never discussed separately in reviews, it is not possible to assess whether results would differ by methodological rigor. Nevertheless, in general, we do not find conclusions to systematically differ, depending on whether non-randomized, qualitative, and/or mixed-method studies were included in the review. In light of the above observations, we did not weigh evidence according to review quality.

Fourth, beyond AMSTAR, a quality issue is whether a meta-analysis has sufficient statistical power to detect intervention effects. Power is likely a concern given the fact that many meta-analyses were attempted on a small number of studies with small samples and heterogenous findings. Although the issue of statistical power is specific to quantitative analysis, the general concern about whether reliable and meaningful findings can be obtained from a small number of studies holds for purely qualitative systematic reviews as well. *Perhaps many published reviews have been premature. Considering also the proliferation of reviews in this field, there is a lot to ponder whether we are sacrificing quality for quantity.*

Lastly, the quality of evidence amassed would be affected by the inconsistent classification of interventions across reviews. This is not an unusual phenomenon, including reviews conducted by the same research group. In fact, given the current practice, it is possible for researchers to publish multiple reviews, each on a selected segment of the literature (e.g., a particular intervention type, a particular delivery medium such as touchscreen, or a particular outcome alone such as burden). When this is done, it is possible for interventions to be classified differently from one review to another, without the practice being obvious to readers. This is especially problematic when done by the same group of researchers, giving the impression that classifications were manipulated to produce certain findings. When the study involved had strong effects, or lack of, it would contribute disproportionally to the pooled effects of different intervention categories [21, 36, 37]. Doing so would certainly bias the overall impression concerning the effects of interventions. At the same time, such a practice is much less likely to happen in a broad coverage of the literature including different intervention types, outcomes, and formats.

## Conclusions and recommendations

We acknowledge that meta-reviews are just one approach to synthesize the literature. For example, a comprehensive meta-analysis that synthesize data at the study level may reveal more (and slightly different) findings [83]. The conclusions possible with this meta-review are limited by the quality and conclusions of the previous reviews. Therefore, methodological issues were taken into account when interpreting findings from the reviews.

In conclusion, depression could be relieved by a variety of interventions, with psychoeducation, counseling/psychotherapy, OT, mindfulness, and multicomponent interventions receiving the strongest support. Evidence of intervention effect was also found for quality of life (psychoeducation), mastery (psychoeducation, occupational therapy and multicomponent interventions) and communication skills (communication training). By contrast, anxiety and social support were by and large not amenable to interventions. There appeared to be a weak intervention effect on burden but it was difficult to pinpoint which interventions were more effective than others. Among the various interventions, support groups and respite were generally ineffective. The current evidence does not support dyadic programs to be superior to caregiver-only programs. There was no conclusive evidence suggesting whether group or individual programs were better.

Whether a program works may well depend on whether its focus matches the needs of the caregiver. Nevertheless, the existing reviews did not support multicomponent interventions to have broader impacts than single-component programs. Given the diversified needs of caregivers along the course of dementia, developing interventions with broader impacts and packaging them to meet caregivers’ changing needs in the long haul should be a priority for researchers and practitioners. This also implies more attention to outcomes other than burden and depression, especially positive outcomes such as mastery and QoL. Other positive outcomes such as positive aspects of caregiving [84, 85] also deserve more attention given their relevance to caregivers’ functioning.

As said, consistency in the language used to classify interventions, or at least transparency in the way classifications were done, is crucial for meaningful results to emerge from reviews. Without such transparency and consistency, it would be difficult to translate review findings into practice recommendations for community or healthcare settings. A more appropriate and comprehensive taxonomy to classify interventions is needed by the field [11] (see Cheng et al. [83] for such an example). To facilitate classification, intervention researchers should provide clear and sufficiently detailed descriptions of different intervention components/modules and how they are put into action. Moreover, for the sake of transparency, review articles should provide similar information about each individual study, albeit in a more succinct way, so that the basis for grouping interventions into a specific category is apparent [11]. For this reason, meta-analytic studies that simply report effect sizes, without making such information available to readers, is less than optimal.

In addition, to provide the field with more informative reviews, we recommend that future reviews should aim to be broader in scope, covering different interventions and outcomes. We need fewer and larger reviews than many small-scale reviews, so that conclusions would be more reliable and robust.

## Data Availability

Data sharing is not applicable to this article as no datasets were generated or analyzed during the current study.

## Abbreviations

AMSTAR: Assessment of Multiple Systematic Reviews
CR: Care-recipient
CBT: Cognitive-behavioral therapy
OT: Occupational therapy
QoL: Quality of life
ROB: Risk of bias

## References

[1] Alzheimer’s Disease International (2015). World Alzheimer report 2015 - the global impact of dementia: an analysis of prevalence, incidence, cost and trends.

[2] Wimo A, Gauthier S, Prince M (2018). Global estimates of informal care.

[3] Cheng S-T (2017). Dementia caregiver burden: a research update and critical analysis. Curr Psychiatry Rep.

[4] Gitlin LN, Hodgson N, Gaugler JE, Kane RL (2015). Caregivers as therapeutic agents in dementia care: the evidence-base for interventions supporting their role. Family caregiving in the new normal Philadelphia.

[5] Clarkson P, Hughes J, Xie C, Larbey M, Roe B, Giebel CM (2017). Overview of systematic reviews: effective home support in dementia care, components and impacts—stage 1, psychosocial interventions for dementia. J Adv Nurs.

[6] Dickinson C, Dow J, Gibson G, Hayes L, Robalino S, Robinson L (2017). Psychosocial intervention for carers of people with dementia: what components are most effective and when? A systematic review of systematic reviews. Int Psychogeriatr.

[7] Huis In Het Veld JG, Verkaik R, Mistiaen P, van Meijel B, Francke AL (2015). The effectiveness of interventions in supporting self-management of informal caregivers of people with dementia: a systematic meta review. BMC Geriatr.

[8] Gilhooly KJ, Gilhooly MLM, Sullivan MP, McIntyre A, Wilson L, Harding E (2016). A meta-review of stress, coping and interventions in dementia and dementia caregiving. BMC Geriatr.

[9] Biondi-Zoccai G (2016). Umbrella reviews: evidence synthesis with overviews of reviews and meta-epidemiologic studies.

[10] Hennessy EA, Johnson BT, Keenan C (2019). Best practice guidelines and essential methodological steps to conduct rigorous and systematic meta-reviews. Appl Psychol Health Well Being.

[11] Gaugler JE, Jutkowitz E, Shippee TP, Brasure M (2017). Consistency of dementia caregiver intervention classification: an evidence-based synthesis. Int Psychogeriatr.

[12] Pinquart M, Sörensen S (2006). Helping caregivers of persons with dementia: which interventions work and how large are their effects?. Int Psychogeriatr.

[13] Shea BJ, Reeves BC, Wells G, Thuku M, Hamel C, Moran J (2017). AMSTAR 2: a critical appraisal tool for systematic reviews that include randomised or non-randomised studies of healthcare interventions, or both. BMJ.

[14] Abrahams R, Liu KPY, Bissett M, Fahey P, Cheung KSL, Bye R (2018). Effectiveness of interventions for co-residing family caregivers of people with dementia: systematic review and meta-analysis. Aust Occup Ther J.

[15] Backhouse A, Ukoumunne OC, Richards DA, McCabe R, Watkins R, Dickens C (2017). The effectiveness of community-based coordinating interventions in dementia care: a meta-analysis and subgroup analysis of intervention components. BMC Health Serv Res.

[16] Bernardo LD, Raymundo TM (2018). Physical and social environment in the occupational therapeutic intervention process for elderly with Alzheimer’s disease and their caregivers: a systematic review of the literature. Braz Jour Occup Ther.

[17] Boots LMM, de Vugt ME, Van Knippenberg RJ, Kempen GI, Verhey FR (2014). A systematic review of internet-based supportive interventions for caregivers of patients with dementia. Int J Geriatr Psychiatry.

[18] Brodaty H, Arasaratnam C (2012). Meta-analysis of nonpharmacological interventions for neuropsychiatric symptoms of dementia. Am J Psychiatry.

[19] Chien L, Chu H, Guo J, Liao Y, Chang L, Chen C (2011). Caregiver support groups in patients with dementia: a meta-analysis. Int J Geriatr Psychiatry.

[20] Clarkson P, Hughes J, Roe B, Giebel CM, Jolley D, Poland F (2018). Systematic review: effective home support in dementia care, components and impacts–stage 2, effectiveness of home support interventions. J Adv Nurs.

[21] Collins RN, Kishita N (2019). The effectiveness of mindfulness- and acceptance-based interventions for informal caregivers of people with dementia: a meta-analysis. Gerontologist.

[22] Cooper C, Balamurali TBS, Selwood A, Livingston G (2007). A systematic review of intervention studies about anxiety in caregivers of people with dementia. Int J Geriatr Psychiatry.

[23] Corbett A, Stevens J, Aarsland D, Day S, Moniz-Cook E, Woods R (2012). Systematic review of services providing information and/or advice to people with dementia and/or their caregivers. Int J Geriatr Psychiatry.

[24] Dam AEH, de Vugt ME, Klinkenberg IPM, Verhey FRJ, van Boxtel MPJ (2016). A systematic review of social support interventions for caregivers of people with dementia: are they doing what they promise?. Maturitas.

[25] Deeken F, Rezo A, Rapp M (2018). Evaluation of technology-based interventions for informal caregivers of patients with dementia – a meta-analysis of randomized controlled trials. Am J Geriatr Psychiatry.

[26] Egan KJ, Pinto-Bruno ÁC, Bighelli I, Berg-Weger M, van Straten A, Albanese E (2018). Online training and support programs designed to improve mental health and reduce burden among caregivers of people with dementia: a systematic review. J Am Med Dir Assoc.

[27] Eggenberger E, Heimerl K, Bennett MI (2013). Communication skills training in dementia care: a systematic review of effectiveness, training content, and didactic methods in different care settings. Int Psychogeriatr.

[28] Elvish R, Lever S, Johnstone J, Cawley R, Keady J (2013). Psychological interventions for carers of people with dementia: a systematic review of quantitative and qualitative evidence. Couns Psychother Res.

[29] Gallagher-Thompson D, Coon DW (2007). Evidence-based psychological treatments for distress in family caregivers of older adults. Psychol Aging.

[30] Godwin KM, Mills WL, Anderson JA, Kunik ME (2013). Technology-driven interventions for caregivers of persons with dementia: a systematic review. Am J Alzheimers Dis Other Dement.

[31] Greenwood N, Pelone F, Hassenkamp A (2016). General practice based psychosocial interventions for supporting carers of people with dementia or stroke: a systematic review. BMC Fam Pract.

[32] Hopkinson MD, Reavell J, Lane DA, Mallikarjun P (2019). Cognitive behavioral therapy for depression, anxiety, and stress in caregivers of dementia patients: a systematic review and meta-analysis. Gerontologist.

[33] Hurley RVC, Patterson TG, Cooley SJ (2014). Meditation-based interventions for family caregivers of people with dementia: a review of the empirical literature. Aging Ment Health.

[34] Jensen M, Agbata IN, Canavan M, McCarthy G (2015). Effectiveness of educational interventions for informal caregivers of individuals with dementia residing in the community: systematic review and meta-analysis of randomised controlled trials. Int J Geriatr Psychiatry.

[35] Jütten LH, Mark RE, Wicherts JM, Sitskoorn MM (2018). The effectiveness of psychosocial and behavioral interventions for informal dementia caregivers: meta-analyses and meta-regressions. J Alzheimers Dis.

[36] Kaddour L, Kishita N, Schaller A (2019). A meta-analysis of low-intensity cognitive behavioral therapy-based interventions for dementia caregivers. Int Psychogeriatr.

[37] Kishita N, Hammond L, Dietrich CM, Mioshi E (2018). Which interventions work for dementia family carers? An updated systematic review of randomized controlled trials of carer interventions. Int Psychogeriatr.

[38] Kor PPK, Chien WT, Liu JYW, Lai CKY (2018). Mindfulness-based intervention for stress reduction of family caregivers of people with dementia: a systematic review and meta-analysis. Mindfulness.

[39] Lamotte G, Shah RC, Lazarov O, Corcos DM (2017). Exercise training for persons with Alzheimer's disease and caregivers: a review of dyadic exercise interventions. J Mot Behav.

[40] Laver K, Milte R, Dyer S, Crotty M (2017). A systematic review and meta-analysis comparing carer focused and dyadic multicomponent interventions for carers of people with dementia. J Aging Health.

[41] Li R, Cooper C, Austin A, Livingston G (2013). Do changes in coping style explain the effectiveness of interventions for psychological morbidity in family carers of people with dementia? A systematic review and meta-analysis. Int Psychogeriatr.

[42] Lins S, Hayder-Beichel D, Rücker G, Motschall E, Antes G, Meyer G (2014). Efficacy and experiences of telephone counselling for informal carers of people with dementia. Cochrane Database Syst Rev.

[43] Liu Z, Chen QL, Sun YY (2017). Mindfulness training for psychological stress in family caregivers of persons with dementia: a systematic review and meta-analysis of randomized controlled trials. Clin Interv Aging.

[44] Llanque SM, Enriquez M (2012). Interventions for Hispanic caregivers of patients with dementia: a review of the literature. Am J Alzheimers Dis Other Dement.

[45] Maayan N, Soares-Weiser K, Lee H (2014). Respite care for people with dementia and their carers. Cochrane Database Syst Rev.

[46] McKechnie V, Barker C, Stott J (2014). Effectiveness of computer-mediated interventions for informal carers of people with dementia—a systematic review. Int Psychogeriatr.

[47] Morris L, Horne M, McEvoy P, Williamson T (2018). Communication training interventions for family and professional carers of people living with dementia: a systematic review of effectiveness, acceptability and conceptual basis. Aging Ment Health.

[48] Nguyen H, Terry D, Phan H, Vickers J, McInerney F (2019). Communication training and its effects on carer and care-receiver outcomes in dementia settings: a systematic review. J Clin Nurs.

[49] Olazarán J, Reisberg B, Clare L, Cruz I, Peña-Casanova J, del Ser T (2010). Nonpharmacological therapies in Alzheimer’s disease: a systematic review of efficacy. Dement Geriatr Cogn Disord.

[50] Orgeta V, Miranda-Castillo C (2014). Does physical activity reduce burden in carers of people with dementia? A literature review. Int J Geriatr Psychiatry.

[51] Parra-Vidales E, Soto-Perez F, Perea-Bartolome MV, Franco-Martin MA, Munoz-Sanchez JL (2017). Online interventions for caregivers of people with dementia: a systematic review. Actas Esp Psiquiatr.

[52] Petriwskyj A, Parker D, O'Dwyer S, Moyle W, Nucifora N (2016). Interventions to build resilience in family caregivers of people living with dementia: a comprehensive systematic review. JBI Database System Rev Implement Rep.

[53] Piersol CV, Canton K, Connor SE, Giller I, Lipman S, Sager S (2017). Effectiveness of interventions for caregivers of people with Alzheimer’s disease and related major neurocognitive disorders: a systematic review. Am J Occup Ther.

[54] Powell J, Chiu T, Eysenbach G (2008). A systematic review of networked technologies supporting carers of people with dementia. J Telemed Telecare.

[55] Rausch A, Caljouw MAA, van der Ploeg ES (2017). Keeping the person with dementia and the informal caregiver together: a systematic review of psychosocial interventions. Int Psychogeriatr.

[56] Schoenmakers B, Buntinx F, DeLepeleire J (2010). Supporting the dementia family caregiver: the effect of home care intervention on general well-being. Aging Ment Health.

[57] Scott JL, Dawkins S, Quinn MG, Sanderson K, Elliott KJ, Stirling C (2016). Caring for the carer: a systematic review of pure technology-based cognitive behavioral therapy (TB-CBT) interventions for dementia carers. Aging Ment Health.

[58] Selwood A, Johnston K, Katona C, Lyketsos C, Livingston G (2007). Systematic review of the effect of psychological interventions on family caregivers of people with dementia. J Affect Disord.

[59] Smith R, Greenwood N (2014). The impact of volunteer mentoring schemes on carers of people with dementia and volunteer mentors: a systematic review. Am J Alzheimers Dis Other Dement.

[60] Smits CHM, de Lange J, Dröes R, Meiland F, Vernooij-Dassen M, Pot AM (2007). Effects of combined intervention programmes for people with dementia living at home and their caregivers: a systematic review. Int J Geriatr Psychiatry.

[61] Tang WK, Chan CYJ (2016). Effects of psychosocial interventions on self-efficacy of dementia caregivers: a literature review. Int J Geriatr Psychiatry.

[62] Thompson CA, Spilsbury K, Hall J, Birks Y, Barnes C, Adamson J (2007). Systematic review of information and support interventions for caregivers of people with dementia. BMC Geriatr.

[63] Tretteteig S, Vatne S, Rokstad AMM (2016). The influence of day care centres for people with dementia on family caregivers: an integrative review of the literature. Aging Ment Health.

[64] Tyack C, Camic PM (2017). Touchscreen interventions and the well-being of people with dementia and caregivers: a systematic review. Int Psychogeriatr.

[65] Vandepitte S, Van den Noortgate N, Putman K, Verhaeghe S, Verdonck C, Annemans L (2016). Effectiveness of respite care in supporting informal caregivers of persons with dementia: a systematic review. Int J Geriatr Psychiatry.

[66] Vandepitte S, Van den Noortgate N, Putman K, Verhaeghe S, Faes K, Annemans L (2016). Effectiveness of supporting informal caregivers of people with dementia: a systematic review of randomized and non-randomized controlled trials. J Alzheimers Dis.

[67] Vernooij-Dassen M, Draskovic I, McCleery J, Downs M (2011). Cognitive reframing for carers of people with dementia. Cochrane Database Syst Rev.

[68] Waller A, Dilworth S, Mansfield E, Sanson-Fisher R (2017). Computer and telephone delivered interventions to support caregivers of people with dementia: a systematic review of research output and quality. BMC Geriatr.

[69] Weinbrecht A, Rieckmann N, Renneberg B (2016). Acceptance and efficacy of interventions for family caregivers of elderly persons with a mental disorder: a meta-analysis. Int Psychogeriatr.

[70] Williams F, Moghaddam N, Ramsden S, De Boos D (2019). Interventions for reducing levels of burden amongst informal carers of persons with dementia in the community. A systematic review and meta-analysis of randomised controlled trials. Aging Ment Health.

[71] Wilson S, Toye C, Aoun S, Slatyer S, Moyle W, Beattie E (2017). Effectiveness of psychosocial interventions in reducing grief experienced by family carers of people with dementia: a systematic review. JBI Database System Rev Implement Rep.

[72] Ying J, Wang Y, Zhang M, Wang S, Shi Y, Li H (2018). Effect of multicomponent interventions on competence of family caregivers of people with dementia: a systematic review. J Clin Nurs.

[73] Parker D, Mills S, Abbey J (2008). Effectiveness of interventions that assist caregivers to support people with dementia living in the community: a systematic review. Int J Evid Based Healthc.

[74] Kwon O, Ahn HS, Kim HJ, Park K (2017). Effectiveness of cognitive behavioral therapy for caregivers of people with dementia: a systematic review and meta-analysis. J Clin Neurol.

[75] Van’t Leven N, Prick AJC, Groenewoud JG, Roelofs PDDM, de Lange J, Pot AM (2013). Dyadic interventions for community-dwelling people with dementia and their family caregivers: a systematic review. Int Psychogeriatr.

[76] Hosaka T, Sugiyama Y (2003). Structured intervention in family caregivers of the demented elderly and changes in their immune function. Psychiatry Clin Neurosci.

[77] Tremont G, Davis JD, Bishop DS, Fortinsky RH (2008). Telephone-delivered psychosocial intervention reduces burden in dementia caregivers. Dementia.

[78] Cheng S-T, Au A, Losada A, Thompson LW, Gallagher-Thompson D (2019). Psychological interventions for dementia caregivers: what have we achieved, what have we learned. Curr Psychiatry Rep.

[79] Cheng S-T, Chan WC, Lam LCW (2019). Long-term outcomes of the benefit-finding group intervention for Alzheimer family caregivers: a cluster-randomized double-blind controlled trial. Am J Geriatr Psychiatry.

[80] Lazarus RS, Folkman S (1984). Stress, appraisal, and coping.

[81] Haley WE, Levine EG, Brown SL, Bartolucci AA (1987). Stress, appraisal, coping, and social support as predictors of adaptational outcome among dementia caregivers. Psychol Aging.

[82] O’Shea E, Timmons S, O’Shea E, Fox S, Irving K (2017). Key stakeholders’ experiences of respite services for people with dementia and their perspectives on respite service development: a qualitative systematic review. BMC Geriatr.

[83] Cheng S-T, Li K-K, Losada A, Zhang F, Au A, Thompson LW (2020). The effectiveness of nonpharmacological interventions for informal dementia caregivers: an updated systematic review and meta-analysis. Psychol Aging.

[84] Cheng S-T, Fung HH, Chan WC, Lam LCW (2016). Short-term effects of a gain-focused reappraisal intervention for dementia caregivers: a double-blind cluster-randomized controlled trial. Am J Geriatr Psychiatry.

[85] Cheng S-T, Mak EPM, Kwok T, Fung HH, Lam LCW. Benefit-finding intervention delivered individually to Alzheimer family caregivers: longer-term outcomes of a randomized double-blind controlled trial. J Gerontol B Psychol Sci Soc Sci. 2019; Epub ahead of print.. 10.1093/geronb/gbz118 31556447

